# Deciphering pancreatic neuroendocrine tumors: Unveiling through circulating small extracellular vesicles

**DOI:** 10.1016/j.heliyon.2024.e29079

**Published:** 2024-04-01

**Authors:** Priya Kumari Gorai, Simran Rastogi, Prahalad Singh Bharti, Shipra Agarwal, Sujoy Pal, Mehar Chand Sharma, Rakesh Kumar, Fredrik Nikolajeff, Saroj Kumar, Neerja Rani

**Affiliations:** a Department of Anatomy, All India Institute of Medical Sciences, New Delhi, India; b Department of Biophysics, All India Institute of Medical Sciences, New Delhi, India; c Department of Pathology, All India Institute of Medical Sciences, New Delhi, India; d Department of GI Surgery, All India Institute of Medical Sciences, New Delhi, India; e Department of Nuclear Medicine, All India Institute of Medical Sciences, New Delhi, India; f Department of Health Science, Lulea University of Technology, Sweden

**Keywords:** Pancreatic neuroendocrine tumors, Small extracellular vesicles, Inhibitor of apoptosis protein, Autophagy

## Abstract

The survival rate over a five-year period for rare pancreatic neuroendocrine tumors (PanNET) is notably lower compared to other neuroendocrine tumors due to late-stage detection, which is a consequence of the absence of suitable diagnostic markers; therefore, there exists a critical need for an early-stage biomarker-specific to PanNETs. This study introduces a novel approach, investigating the impact of small extracellular vesicles (sEV) in PanNET growth and metastasis. As proof of concept, this study shows a correlation between sEV concentration in controls and PanNET. Notably, higher sEV concentrations were observed in PanNETs than in controls (p < 0.0001) with a sensitivity of 100%. Further, apparent differences were observed in the sEV concentrations between controls and grades 1 PanNET (p = 0.005). The expression of sEV markers was confirmed using CD63, TSG101, CD9, Flotillin-1, and GAD65 antibodies. Additionally, the expression of cancer marker BIRC2/cIAP1 (p = 0.002) and autophagy marker Beclin-1 (p = 0.02) were observed in plasma-derived sEVs and PanNET tissue. This study represents the first to indicate the increased secretion of sEV in PanNET patients' blood plasma, proposing potential function of sEV as a new biomarker for early-stage PanNET detection.

## Highlights

• Pancreatic neuroendocrine tumors (PanNET) are uncommon, with low survival.
• Early-stage detection techniques are required for improved diagnosis.
• Study observed increased plasma sEVs in PanNET patients than in controls.
• Increased expressions of antiapoptotic and autophagy proteins as sEVs cargo.
• These findings highlight the potential of sEVs in early diagnosis for PanNET.

## Graphical abstract

**Figure:**
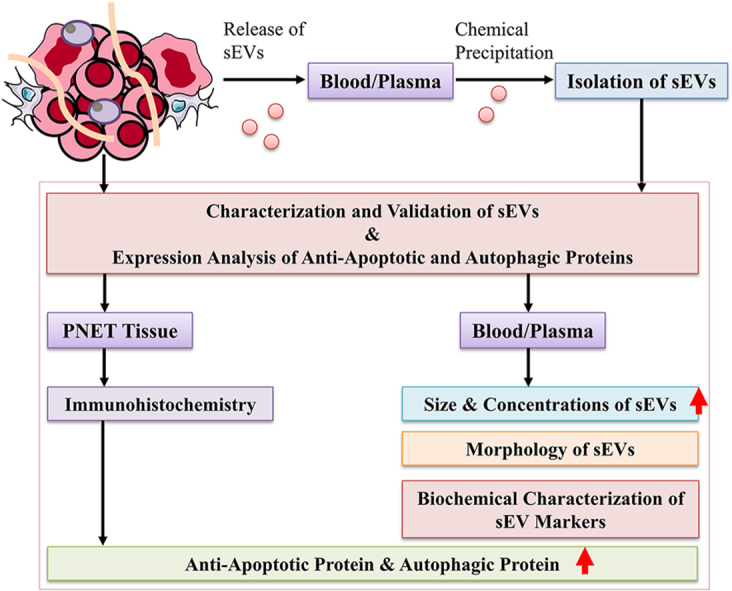
Graphical Abstract.

## 1 Introduction

Pancreatic neuroendocrine tumors (PanNET), an uncommon and diversified class of tumors originating from the endocrine cells of the pancreas, are also called islet cell tumors and have a low incidence rate. However, over the last 4 decades, their incidence has increased sevenfold, with a prevalence of 25–30 individuals per 100,000 population in the USA [[1], [2], [3], [4], [5]]. While heredity is a contributing factor, a individual's diabetes history, pancreatic cancer in the family history, or smoking are additional risk factors. Furthermore, a strong correlation exists between PanNET and a first-degree family history of oesophageal cancer. Additionally, there is a possible connection between first-degree relatives of other gastrointestinal neuroendocrine tumors and sporadic PanNETs. The symptoms of PanNET are indistinguishable from other neuroendocrine tumors and have a poor prognosis with low five-year survival [5,6]. This poor prognosis depends on various factors, like initial detection at the advanced stages since diagnostic imaging methods aren't widely utilized and there are no specific biomarkers for diagnosis. Additionally, poor response to traditional therapy, a high chance of distant metastases, and the only available curative treatment, i.e., surgical excision, are factors that further add to this poor prognosis of PanNET [7,8]. Despite surgical resection, the complete elimination of PanNET remains challenging. Efficient early diagnostic biomarkers are crucial for improving PanNET diagnosis and prognosis, thus reducing the healthcare burden.

In the previous 20 years, scientific and clinical research have advanced significantly to discover novel diagnostic, prognostic, and therapeutic approaches for PanNET. Small extracellular vesicles (sEV) are nanoscale (30–150 nm) vesicles derived from variety of cells types, comprising malignant cells, and have an essential role in the removal of cellular waste and in facilitating intercellular communication. sEVs represent the molecular status of their origin cells and have an essential role in pathophysiology of various diseases [9,10]. sEVs are also widely distributed in bodily fluids such ascites, blood, saliva, and urine and could potentially discover new diagnostic, prognostic, and predictive cancer biomarkers [[11], [12], [13], [14]].

Autophagy and apoptosis pathways perform vital roles in genesis of tumors, its progression, and cancer cells survival [15,16]; therefore, this study examines an autophagy marker and an antiapoptotic protein in PanNET. Studies have found that some Inhibitors of Apoptosis protein (IAPs) like XIAP, cIAP1, cIAP2, and survivin are released into the extracellular spaces by tumor and normal cells via sEVs and are uptaken by neighboring tumor microenvironments as a preventative measure against cell death [17]. Consequently, the differential expressions of IAPs and autophagic proteins were reported in different cancers [18,19]. Baculoviral IAP Repeat Containing 2/cellular IAP1 (BIRC2/cIAP1), one of the members of the IAPs family, has a major impact on the fate and functioning of cells. It influences the signaling of nuclear factor κβ (NF-κβ) and suppresses apoptosis [19,20]. Beclin-1, an essential autophagy protein, suppresses tumor formation by removing defective or damaged organelles in healthy cells and supports tumorigenesis by stimulating cancer initiation and progression [21]. Research has revealed that decreased Beclin-1 expression was found in certain cancers like glioblastomas, ovarian, hepatocellular, and oesophageal cancers [22]. Conversely, colorectal, gastric cancer and pancreatic ductal adenocarcinoma cells have shown increased expression [22,23], whereas, in adjacent noncancerous tissues, little to no Beclin-1 expression was observed, implying tissue-specific functions for Beclin-1.

Given the rare and low-incidence nature of PanNET and its notably low five-year survival rate among neuroendocrine tumors, this preliminary research with a relatively small sample size is sufficient for a proof-of-concept. The study establishes a link between sEV concentration and PanNET grades, with the presence of inhibitors of apoptotic protein (BIRC2/cIAP1) and Beclin-1 in plasma-derived sEV cargo. While there is limited research on the role of plasma-derived sEVs in PanNET, this field is still in its early stages; therefore, this work aims to propose a clinically acceptable, cost-effective with high specificity and sensitivity early detection technique based on plasma derived sEVs.

## 2 Material and methods

### 2.1 Subjects

51 subjects were included in the study, including 26 PanNET patients and 25 controls. Patient samples were collected between December 2020 and February 2022, with signed informed consent and institutional ethics committee approval (IECPG-452/25.08.21: Number of Ethical Approval) at All India Institute of Medical Sciences, New Delhi, India. Blood samples were taken from 26 clinically proven PanNET patients (Functional PanNET) without treatment and 20 healthy controls. The tissue samples were acquired from 07 PanNET patients who underwent surgery and their adjacent healthy tissue as controls (n = 05). Due to the rarity and poor prognosis of PanNET, a small sample size was considered sufficient for this preliminary investigation to establish a proof-of-concept for the effective detection of PanNET using sEVs (small extracellular vesicles) at an early stage. Hormonally active PanNET patients of any sex and age group, with histologically and FNAC (Fine needle aspiration cytology) confirmed PanNET and different grades based on the Ki67 and Mitotic indexes, were recruited from the Nuclear Medicine and Gastrointestinal Surgery departments of AIIMS, New Delhi. Grade 1 PanNETs had a ≤3% Ki67 index and <2/10 high-power field (HPF) mitotic count, whereas Grade 2 PanNETs exhibited a mitotic count of 2–20/10 HPF and a Ki67 score of 3–20% [23,24]. The control group consisted of individuals (n = 20) who did not have diabetes and had not had any medical procedures, including radiation, chemotherapy, surgery, or peptide receptor therapy [25]. A detailed patient history is presented in Table 1.

**Table 1:** Demographic details of PanNET patients and healthy controls.

Characteristics		Plasma Sample		Tissue Sample^c^	
		PanNET Patients (n = 26)	Controls^a^ (n = 20)	PanNET Patients (n = 07)	Controls^b^ (n = 05)
Sex	Male	15	11	05	03
	Female	11	09	02	02
Age (in years)	Mean ± SEM	42.6 ± 2.73	34.95 ± 2.23	33.2 ± 3.8	39.8 ± 6.5
	Range	20–70	25–57	20–49	26–49
Tumor Grading	Grade 1	13	–	04	–
	Grade 2	13	–	03	–
Ki67 Values	1–3%	13	–	04	–
	3–5%	10	–	02	–
	>5%	03	–	01	–
Distant Metastasis		12	–	00	–

a Plasma from healthy controls was used.

b Adjacent (healthy tissue) pancreatic tissue from PanNET patients was used as a control.

c Out of 26 PanNET patients enrolled, only 7 PanNET patients underwent surgery.

### 2.2 Samples collection

In the resting condition, 5 ml of peripheral blood were extracted from healthy individuals as well as PanNET patients. In order to extract the plasma, the blood samples were centrifuged for 10 min at 4 °C at 2000 rpm. [25], and the supernatant was collected. Finally, the plasma samples were then kept at −80 °C until more research was conducted.

### 2.3 Plasma sEV isolation

From the cleared plasma samples, sEV were separated using the chemical precipitation method by mixing the plasma with 14% polyethylene glycol, it was then incubated at 4 °C for 6–8 h. After an hour of centrifuging the mixture at 13,000G, the pellet was resuspended in 1 × phosphate buffer saline [26].

### 2.4 Transmission electron microscopy-based morphological characterization

For Transmission electron microscopy, using carbon-coated copper grids, the sEV samples were adsorbed. (01843, Ted Pella), followed by using 2% aqueous uranyl acetate as a negative stain for 15 s. The grids were examined under a transmission electron microscope after being blot-dried. (Tecnai G2, FEI).

### 2.5 Nanoparticle tracking analysis

sEV quantification was carried out via nanoparticle tracking analysis (NTA) (Germany, Particle Matrix) in three modes: Scatter mode (1:1000 dilution), Fluorescent lipid-binding dye-labeled sEV (1:500), and antibody-labeled sEV (1:200) in 1 × phosphate-buffer saline. For analysis, a volume of 0.5 ml was injected into the NTA apparatus. All dilutions were made in 1 × phosphate buffer saline. For Scatter mode, In 1 × PBS buffer, isolated samples of sEV were evaluated at 1:1000 dilutions. For fluorescent mode, sEVs were labeled with a plasma membrane dye (Invitrogen, CellMask™ Deep Red, C10046) in a 1:1000 dye-to-sample ratio and analyzed using a laser of 640 nm. In the antibody-based mode, sEVs were tagged using the R&D Systems-produced CD63 Alexa fluor 488 antibody (IC5048G; stock concentration: 0.2 mg/ml). for 2 h at room temperature with a 1:10 antibody-to-sample ratio. Moreover, the antibody-labeled sEVs were investigated in fluorescence mode using a 488 nm laser in the Zeta View Twin system.

### 2.6 Western blot profiling

sEV validation involved western blotting with Invitrogen's anti-CD63 (10628D), anti-TSG101 (MA-1-23296, Invitrogen), anti-CD9 (Invitrogen, PA5-86534), anti-Flotillin-1 (Invitrogen, PA5-17127), calnexin (E-AB-14819; Elabsciences), and anti-GAD65 (a marker for islets origin sEVs, PA5-22260, Invitrogen) antibodies. As the internal control, β-actin was used. The ImageJ program was used to quantify band intensities (NIH, USA). sEV cargo proteins, BIRC2/cIAP1 (DF6167, Affinity Bioscience), and Beclin-1 (E-AB-53242, Elabsciences) were assessed. sEVs were lysed through ultrasonication, and The Bicinchoninic Acid test (22802, ThermoFisher Scientific) was used to quantify total protein with BSA as the protein standard. Isolated sEV samples (10 μg) from control and patients were transferred to BioRad nitrocellulose membranes after being separated by 10% SDS PAGE. Further, Membranes were blocked in 3% BSA in TBS-T before primary antibodies (CD63, TSG10, anti-CD9, Flotillin-1, calnexin, GAD65, BIRC2/cIAP1, and Beclin-1) were added for overnight at 4 °C, further HRP-tagged secondary antibody (at room temperature for 2 h in the dark) was used. Nitrocellulose membranes were stained using the G-biosciences' Femto LUCENT™ PLUS-HRP kit (786-003) for HRP-based electroluminescence and imaged using a GEL-Doc apparatus (Azure Biosystems).

### 2.7 Immunohistochemistry

IHC was performed to assess the expression of BIRC2/cIAP1 and Beclin-1. Sections (5–6 μm thick) were cut with a microtome, de-paraffinized using xylene, hydrated with alcohol, and rinsed with water. Antigen retrieval involved incubating the section in a water bath with citrate buffer (pH 6.0) for 30 min at 98 °C, followed by washing with PBST. Endogenous peroxidases were blocked with 3% H2O2 in methanol for 10 min at RT. Blocking serum buffer (5% bovine serum albumin) was applied for 20 min at room temperature. Primary antibodies for BIRC2/cIAP1 (1:100, DF6167, Affinity Bioscience) and Beclin-1 (1:100, E-AB-53242, Elabsciences) were added overnight at 4 °C, further HRP-tagged secondary antibody (at RT in the dark for 2 h) was used. 3,3′-Diaminobenzidine (DAB) was used as the chromogen substrate, and sections were dehydrated and mounted on glass slides. Visualization and analysis were performed using a Nikon Eclipse TiS microscope (Nikon Instruments, USA) with NIS element AR software. BIRC2/cIAP1 and Beclin-1 expression were determined through immunohistochemistry using DAB as a chromogen. The brown color indicated positive staining and various intensities were measured. 10 Individual high-power fields were randomly selected and examined by two independent observers for semi-quantitative assessment. The intensities in these areas were recorded on a scale of 0 for no staining, + for mild staining, ++ for moderate staining, and +++ for severe staining.

### 2.8 Statistical analysis

statistical analysis of the data was performed by utilizing 8.0 version of GraphPad Prism where One-way ANOVA was used to compare the three categories of subjects, and the multiple comparisons between all the categories were achieved through the Kruskal-Wallis test. The unpaired *t*-test was done to compare controls and PanNET patients, p < 0.05 as the threshold for significance. However, during statistical analysis, the data did not follow a normal distribution because of outliers and a small sample size. Non-parametric tests were therefore used. When assessing test accuracy, the receiver operating characteristic (ROC) curve is crucial. The AUC (Area Under the Curve) indicates how well the test can distinguish between different groups. Plotting TPR (sensitivity) vs FPR (1-specificity), with TPR on the y-axis and FPR on the x-axis, will show this visually. A test that is better is closer to an AUC of 1, which denotes exceptional separability, while a test that is poorer is closer to an AUC of 0, which denotes the lowest separability. For sEV concentrations in PanNET patients and controls, ROC curve analysis measures sensitivity and specificity, represents separability between classes, and assesses test accuracy using AUC.

## 3 Results

In this study, hormonally active PanNET patients of any sex and age group were recruited. The PanNET patients mean age was 42.6 ± 2.73 years (mean ± SEM), and controls were 34.95 ± 2.23 years (mean ± SEM). The age range of PanNET patients was 20–70 years, and controls were 25–57 years. The grading of PanNETs was done according to the 2017 WHO criteria [27]. Grade 1 & 2 tumors were observed in 50-50% (13/26, each), and 46.1% of PanNET patients (12/26) had shown distant metastasis. The PanNET patient and healthy control information are summarized in Table 1. An appropriate sample size (n = 51) for a proof-of-concept study is provided by the extremely low incidence rate of PanNET.

### 3.1 sEVs in differentiating PanNET from controls

**Fig. 1:**
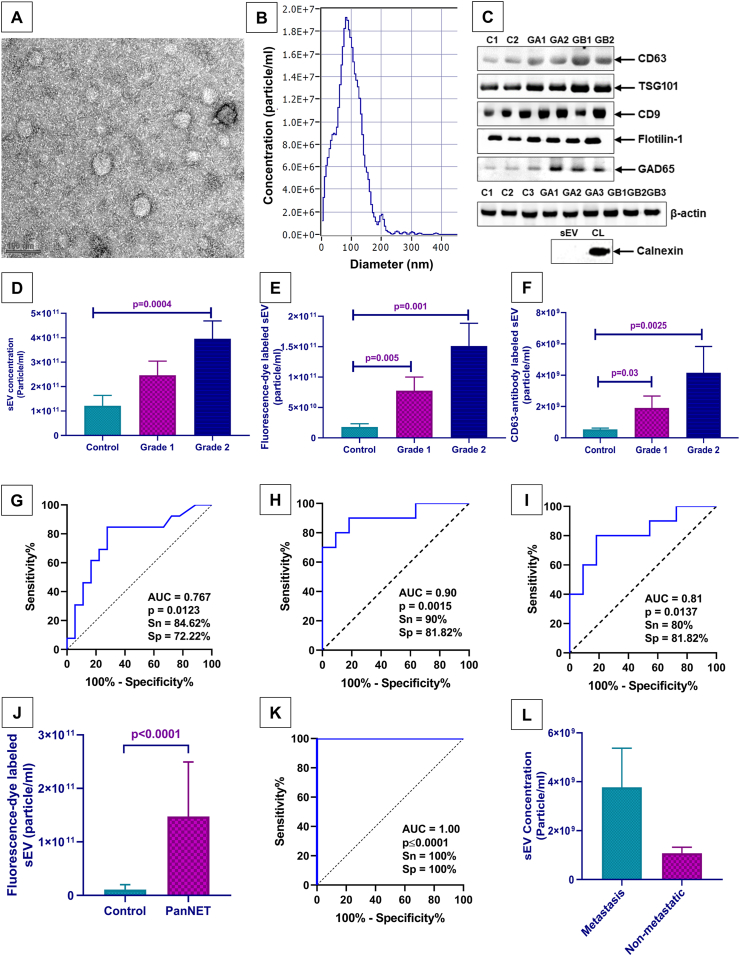
**small Extracellular Vesicles (sEVs) Characterization and Validation** (A) TEM image of isolated plasma sEV (scale = 100 nm); (B) Graphical representation of scatter mode NTA with a concentration in particle/ml and size in nm (approx. 90% of the particles are in the range of 40–150 nm). (C) sEV presence using several exosomal markers in all three categories control (C1, C2) and PanNETs patients [Grade 1 (GA1, GA2) & 2 (GB1, GB2)]. sEV: small extracellular vesicles; CL: cell lysate. (D) The sEV levels in scatter mode of NTA between controls, Grade 1 & 2 (p = 0.0004). (E) The fluorescent-tagged sEV levels between controls, Grade 1 & 2 (p = 0.001, 0.005). (F) CD63-antibody labeled sEV levels between controls, Grade 1 & 2 (p = 0.0025, 0.03). ROC curve analysis for sEV levels in controls vs PanNET patients in (G) scatter mode (p = 0.0123) NTA, (H) fluorescence mode (p = 0.0015) NTA, and (I) antibody mode (p = 0.0137) NTA, with their AUC (area under the curve), Sn (sensitivity), and Sp (specificity) in respective figures. (J) The fluorescent-tagged sEV levels between controls and PanNET patients (p < 0.0001). (K) ROC curve analysis for sEV levels in controls vs PanNET patients and (L) metastasis & non-metastasis in PanNET patients.

Nanoparticle tracking analysis (NTA), Transmission electron microscopy (TEM), and western blotting protein expression analysis were done to characterize and validate the plasma derived sEV. In TEM, the negatively stained isolated plasma sEV appeared round with a distinctive lipid bilayer membrane (Fig. 1A). Further, The concentration of the isolated sEVs (particles/ml) and their size (in nm) were measured using NTA. (Table 2). Fig. 1B is the representative graph of the peak analysis of NTA, showing more than 90% of the particles between 40 and 150 nm diameter. The confirmation of sEV presence was accomplished using exosomal markers (Fig. 1C). Subsequently, the concentration of sEVs was assessed through nanoparticle tracking analysis. The quantification of sEVs concentration in scatter mode shows a higher concentration of sEV in PanNET patients in Grades 1 & 2 than controls (p = 0.0004) (Fig. 1D). When sEV concentration in the NTA's scatter mode was analyzed using a ROC curve for PanNET patients and controls, AUC = 0.76 (p = 0.0123) was obtained, indicating 84.6% sensitivity and 72.22% specificity. (Fig. 1G). The sEV tagged with fluorescent dye are significantly higher (p = 0.001) in Grades 1 & 2 in comparison to controls; also, the sEV concentration in Grade 1 is significantly higher than the controls (p = 0.005) (Fig. 1E). Additionally, ROC curve analysis was performed to determine the sEV concentration in fluorescent mode NTA for PanNET patients and controls. The results showed an AUC of 0.90 (p = 0.0015), 90% sensitivity, and 81.82% specificity (Fig. 1H). In the antibody mode of NTA, a higher concentration of CD63-labeled sEVs was observed in Grade 1 and Grade 2 compared to controls (p = 0.0025). Also, the sEV concentration in Grade 1 is significantly higher than the control (p = 0.03) (Fig. 1F). When the sEV concentration in antibody mode NTA for PanNET patients and controls was analyzed using the ROC curve, the results showed an AUC of 0.81 (p = 0.0137), 80% sensitivity, and 81.82% specificity (Fig. 1I). In all three NTA modes, grade 1 and grade 2 PanNET patients had higher sEV concentrations than healthy controls (Fig. 1D, E & F).

**Table 2:** NTA Analysis of isolated sEVs in PanNET patients and controls (Mean ± SEM).

Subjects	Scatter mode (1:1000 dilution)	Fluorescent dye-labeled exosomes (1:500)	Antibody-labeled exosomes (1:200)
Grade 2 PanNETs	3.96 × 10^11^ ± 7.29 × 10^10^	1.51 × 10^11^ ± 3.73 × 10^10^	4.02 × 10^9^ ± 1.71 × 10^9^
Grade 1 PanNETs	2.46 × 10^11^ ± 5.79 × 10^10^	7.76 × 10^10^ ± 2.24 × 10^10^	1.77 × 10^8^ ± 7.82 × 10^8^
Control	1.21 × 10^11^ ± 4.24 × 10^10^	1.79 × 10^10^ ± 5.31 × 10^9^	9.46 × 10^8^ ± 3.73 × 10^8^
*p* value (Kruskal-Wallis test)	0.0004	0.001	0.0025

Furthermore, a notable difference appeared upon comparing sEV concentrations between patients with PanNET and healthy individuals. Specifically, the sEV concentration within PanNET patients was significantly higher than healthy controls, as evidenced by the fluorescence mode of NTA (p < 0.0001) (Fig. 1J). This distinction yielded an AUC of 1.00 (p < 0.0001), accompanied by a sensitivity and specificity both amounting to 100% (Fig. 1K). However, when examining the impact of metastasis, it was discovered that patients at the metastasis stage exhibited an increased concentration of sEVs compared to those without metastasis (Fig1L). This finding could explain the absence of significant differences in sEVs between Grade 1 and Grade 2 PanNET, as metastasis may be crucial in influencing sEV levels.

### 3.2 sEV markers proteins in PanNET progression

**Fig. 2:**
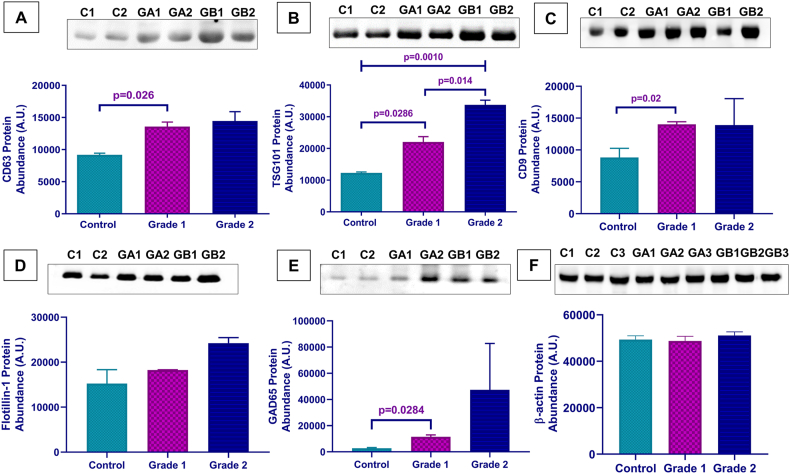
**Expression Analysis of sEV surface marker proteins.** Western blot profiling and densitometric analysis of (A) CD63 (p = 0.026); (B) TSG101 protein (p = 0.0010, 0.0286, 0.0141); (C) CD9 Protein (p = 0.02); (D) Flotillin-1 and (E) GAD65 (p = 0.0284) (F) β-actin.

The validation of sEV through western blotting against sEV protein markers was carried out [28]. We used sEV surface markers CD63, TSG101, CD9, and Flotillin-1 (Fig. 2A, B, C & D; Supplementary Material Figs. S1–S4) Calnexin as a negative marker for sEVs (Fig. 1C; Supplementary Material Fig. S7), and GAD65 as a marker for sEVs originated from islet cells (Fig. 2E–Supplementary Material Fig. S5). The band intensities' densitometry demonstrates a progressive increment of CD63 in Grade 1 and 2 PanNET patients compared to controls, with a significantly higher CD63 expression in Grade 1 PanNET patients than controls (p = 0.02) (Fig. 2A). Densitometry of TSG101 shows a significant increment from controls to Grades 1 & 2 (*p*-value = 0.0010); grade 1 shows significantly higher TSG101 protein abundance than healthy controls (p = 0.02), and the sEV concentration in Grade 2 is significantly higher than Grade 1 (p = 0.0141) (Fig. 2B). Densitometry analysis of the band intensities indicates a progressive increment of CD9 and Flotillin-1 in Grade 1 and 2 PanNET patients compared to controls (Fig. 2C and D). In the case of GAD65, a thin band was observed in the controls. In contrast, band intensity was higher in Grade 1 and Grade 2 PanNET patients. Also, the sEV concentration in Grade 1 is significantly higher than in controls (p = 0.0284), which confirms the increased secretion of sEVs in PanNET compared to controls (Fig. 2E). β-actin was used as an internal control for sEVs in PanNETs and control (Fig. 2F–Supplementary Material Fig. S6).

### 3.3 sEV cargo proteins in PanNET progression

**Fig. 3:**
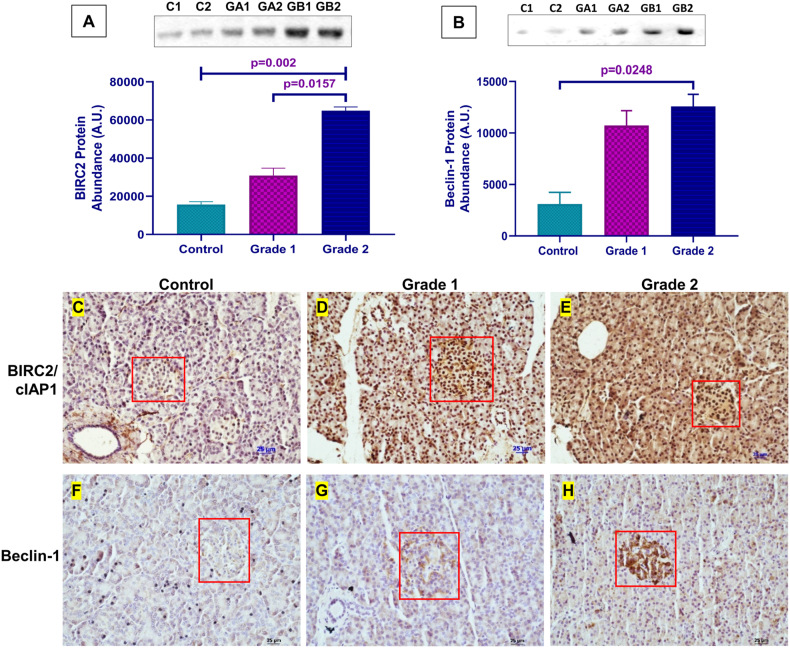
**Expression Analysis of sEV cargo marker proteins.** Western blot profiling and densitometric analysis of (A) BIRC2/cIAP1 (p = 0.002, 0.0157); and (B) Beclin-1(p = 0.0248) in all three categories control (C1, C2) and PanNETs patients [Grade 1 (GA1, GA2) & 2 (GB1, GB2)]. Immunohistochemical expression of BIRC2/cIAP1 and Beclin-1 in controls (C & F, respectively), the red box shows the islet of Langerhans. Immunohistochemical expression of BIRC2/cIAP1 and Beclin-1 in Grade 1 (D & G, respectively) & Grade 2 (E & H, respectively), and the red boxes show tumor sites at the islet of Langerhans in PanNET patients. The scale bar is 25 μm.

Apoptosis and autophagy play crucial roles in tumor development. BIRC2/cIAP1 and Beclin-1 expression were determined through immunohistochemistry, where brown staining in tumor cells signified positive staining, with varying intensities measured in the tissue sections. In this study, the BIRC2 protein expression was observed in the nucleus and cytoplasm of PanNET cells, while the Beclin-1 protein expression was seen in the cytoplasm. The intensity of BIRC2 and Beclin-1 protein expression was mild (+) in Controls (Fig. 3 C & F), moderate (++) in grade 1 tumors (Fig. 3 D & G), and high (+++) in grade 2 tumors (Fig. 3E and H), indicating weak protein expression in controls and strong protein expression in PanNET tumors. Thus, Immunohistochemistry analysis of tumor sites revealed higher expressions of BIRC2/cIAP1 and Beclin-1 in PanNET patients compared to healthy controls. Moreover, there was a noticeable difference in expression between grade 2 and grade 1 PanNET patients, indicating a distinction between tumor grades. Furthermore, we examined the presence of these proteins in the plasma sEV cargo. The expression of BIRC2/cIAP1 was significantly higher in PanNET patients compared to controls (p = 0.001). Notably, there was a significant difference in expression between Grade 2 and Grade 1 PanNETs, with higher expression in Grade 2 (p = 0.015) (Fig. 3A–Supplementary Material Fig. S8). Similarly, the expression of Beclin-1 was higher in patients than in controls (p = 0.0248) (Fig. 3B–Supplementary Material Fig. S9). These findings highlight the increased secretion of plasma-derived sEVs in the blood of PanNET patients and the presence of BIRC2/cIAP1 and Beclin-1 in the sEV cargo, demonstrating significant differences between patients and controls.

## 4 Discussion

Pancreatic neuroendocrine tumors (PanNETs) encompass a range of histopathological variations, including benign to highly malignant tumors. The global incidence of PanNETs has risen over the past four decades, primarily due to the insufficiently precise and sensitive biomarkers and limited accessibility to costly diagnostic imaging techniques, particularly in low-income countries [7,8]; therefore, easy and cost-effective biomarkers for screening and diagnosing pancreatic neuroendocrine tumors are essential.

Small extracellular vesicles (sEVs), characterized by nanoscale dimensions and lipid bilayer envelopes, are discharged by diverse cell types under physiological and pathological conditions. These vesicles play a crucial role in facilitating the transfer of biological information between cells [29]. In the last 20 years, research has shown that the significance of sEVs in the pathophysiology, diagnosis, and treatment of diverse cancers, including prostate, pancreas, breast, ovarian, glioblastoma, melanoma, and others [30,31].

The premetastatic niche, a pre-existing microenvironment shaped by sEVs released from the primary site to distant metastatic sites, has been extensively examined in various tumors. Growing evidence suggests that sEVs play a crucial role as mediators of metastasis [[31], [32], [33]]. Several preclinical and in vitro models have shown that transferring biomolecular cargo between tumor and normal cells via sEVs enhances distal microenvironments by creating an environment conducive to cancer growth [34]. Cancer-derived sEVs can be detected in multiple body fluids, including blood, saliva, urine, and ascites. Isolation and characterization of sEVs can enhance our understanding of cancer pathophysiology and contribute to developing innovative diagnostic and therapeutic strategies [11,30]. In this study, we observed an increased secretion of plasma-derived sEV in PanNET patients than controls using different modes of nanoparticle tracking analysis, such as scatter mode (p = 0.0004), fluorescence mode (p = 0.001), and antibody mode (p = 0.0025). Also, the increase in sEV concentration is observed in grade 1 PanNETs compared to controls. It was also observed that PanNET patients at the metastatic stage exhibited an increased concentration of sEVs compared to those without metastasis. Therefore, utilizing the three modes of NTA for sEV concentration, we could distinguish different grades of PanNET patients from healthy controls. Further, we observed the diagnostic applicability of sEV concentration in differentiating healthy controls to PanNET patients using ROC curve analysis. The area under the curve (AUC), calculated from ROC curve analysis, has a meaningful interpretation for disease classification from healthy subjects. Hence, the ROC curve indicates that the concentration of plasma-derived sEVs has the potential to distinguish PanNET patients from healthy controls [35,36]. In our observation, all three modes of NTA show acceptable AUC values: scatter mode AUC = 0.76 (p = 0.0123), fluorescence mode AUC = 0.9 (p = 0.0015), and antibody mode AUC = 0.81 (p = 0.0137), represents suitability of sEV concentration in diagnosis of PanNET.

Further, to determine the origin of sEVs, we employed GAD65, an intracellular membrane protein found in pancreatic islet cells [[37], [38], [39]], and observed differential expressions in healthy controls and PanNET patients. The presence of GAD65 in plasma-derived sEVs confirms the origin of islet cells-derived sEVs, with increased expression in grade 1 PanNET patients compared to healthy controls. Additionally, sEVs can influence the expression of both autophagy and apoptosis in various diseases. Studies have shown that sEVs have the ability to control Beclin1 expression by paracrine activity, and the specific impact and mechanisms of this regulation can vary depending on the nature and stage of the disease [40,41]. In this study, we noted the existence of inhibitors of apoptotic protein (IAP), BIRC2/cIAP1, and Beclin-1, an autophagy marker, in the cargo of sEVs derived from plasma. IAPs are well recognized for their ability to inhibit caspase and are found to promote tumorigenesis in humans [18,42,43]. In addition to there anti-apoptotic properties, studies have revealed that IAPs regulate autophagy [19]. In general, autophagy deregulation accelerates the growth of tumors. Autophagy also shields poorly vascularized tumors from stress-related cell death, such as starvation and hypoxia, and provides additional survival signals to cancer cells [[44], [45], [46]]. Our study examined the expression of BIRC2/cIAP1 and Beclin-1 to investigate the connection between IAPs and autophagy. We analyzed the expressions through immunohistochemistry and assessed their presence in the cargo of plasma-derived sEVs in both PanNET patients and healthy controls. We observed a progressive increase in BIRC2/cIAP1 and Beclin-1 expression from healthy controls to Grade 1 and Grade 2 PanNET. This study has some limitations that could be addressed in future investigations. Firstly, PanNET with grade 3 tumors was not included, and the sample size was relatively small. The study primarily focused on patients with functional PanNETs so that the findings may vary for non-functional PanNETs.

## 5 Conclusion

These findings suggest that the elevated concentration of plasma sEVs in PanNET could serve as an early disease screening indicator. Moreover, increased expression of BIRC2/cIAP1 and Beclin-1 in the sEV cargo provides additional evidence for their potential as significant biomarkers for PanNET detection. In addition to the early screening of PanNET, sEVs may be a potential tool in assessing tumor aggressiveness, detecting progression or recurrence, and evaluating treatment response; however, these aspects need further evaluation. Understanding these applications comprehensively would enhance our understanding of PanNET behaviour, paving the way for more personalized and effective management strategies.

## Funding source

The research was funded by the Department of Science and Technology, Govt of India (Grant Number: EEQ/2018/000697).

## Data availability

The data that support the findings of this study are available from the corresponding authors, [SK, NR], upon reasonable request.

## Ethics

This study was approved by the Institutional Ethics Committee, All India Institute of Medical Sciences, New Delhi, India (Ethical Approval Number: IECPG-452/25.08.21).

## CRediT authorship contribution statement

**Priya Kumari Gorai:** Writing – review & editing, Writing – original draft, Visualization, Validation, Methodology, Investigation, Formal analysis, Data curation. **Simran Rastogi:** Writing – review & editing, Validation, Methodology, Formal analysis, Data curation. **Prahalad Singh Bharti:** Writing – review & editing, Visualization, Validation, Supervision, Formal analysis. **Shipra Agarwal:** Writing – review & editing, Supervision, Resources. **Sujoy Pal:** Writing – review & editing, Supervision, Resources. **Mehar Chand Sharma:** Writing – review & editing, Supervision, Resources. **Rakesh Kumar:** Writing – review & editing, Supervision, Resources. **Fredrik Nikolajeff:** Writing – review & editing, Supervision. **Saroj Kumar:** Writing – review & editing, Writing – original draft, Visualization, Supervision, Methodology, Funding acquisition, Formal analysis, Conceptualization. **Neerja Rani:** Writing – review & editing, Writing – original draft, Visualization, Validation, Supervision, Methodology, Funding acquisition, Formal analysis, Conceptualization.

## Declaration of competing interest

The authors declare that they have no known competing financial interests or personal relationships that could have appeared to influence the work reported in this paper.
