# Supplementary material for: Deciphering pancreatic neuroendocrine tumors: Unveiling through circulating small extracellular vesicles
Source: Heliyon. 2024 Apr 1;10(7):e29079. doi: 10.1016/j.heliyon.2024.e29079 (PMC11002672; doi:10.1016/j.heliyon.2024.e29079)
Supplement: Multimedia component 1 [file mmc1.docx]

**SUPPLEMENTARY MATERIAL (Fig. S1-S9)**

**
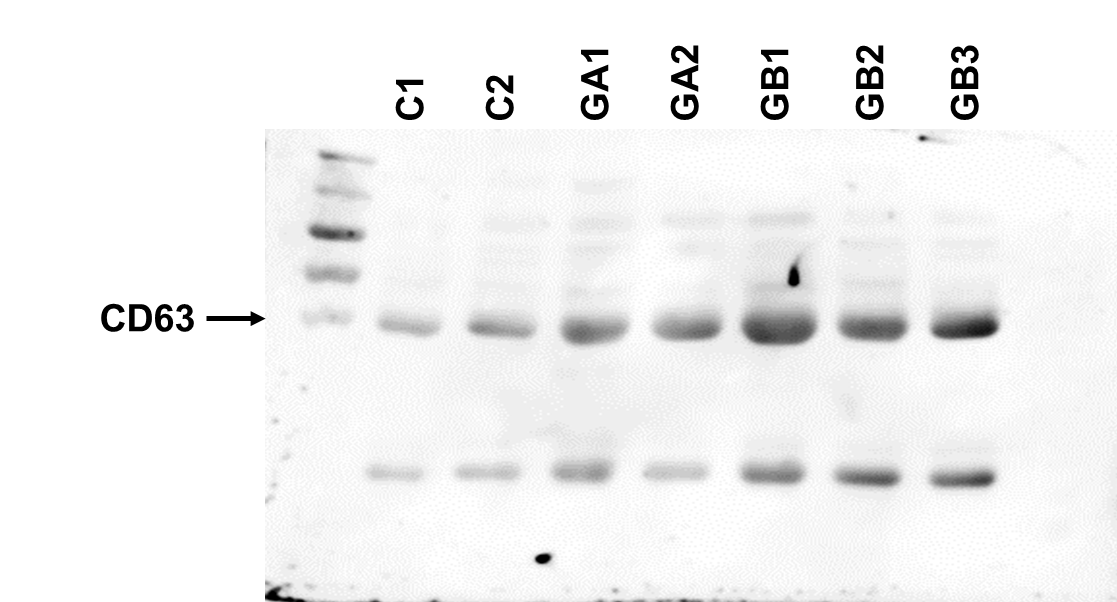
**

**Figure S1: Western blot of anti-CD63** **in Healthy Controls (C1 and C2), Grade I PanNET (GA1 and GA2), Grade II PanNET (GB1, GB2 and GB3).**

**
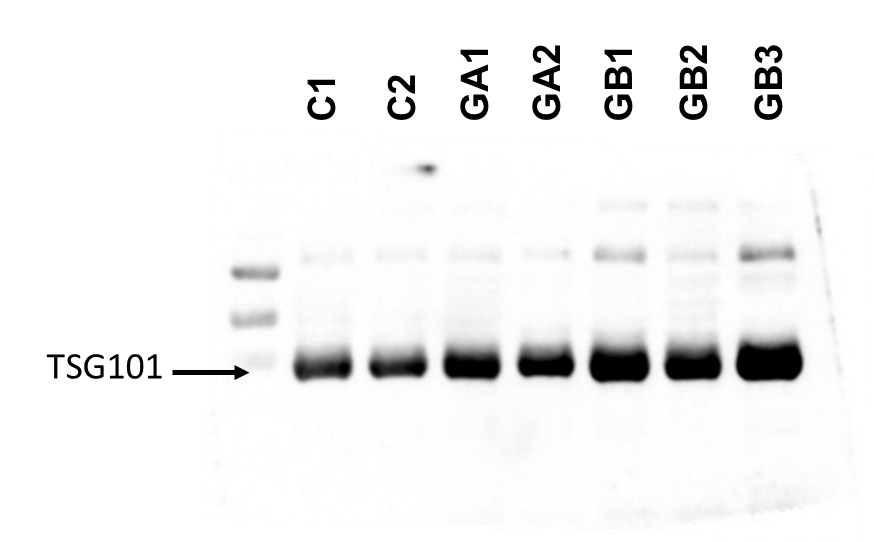
**

**Figure S2: Western blot of anti-TSG101 in Healthy Controls (C1 and C2), Grade I PanNET (GA1 and GA2), Grade II PanNET (GB1, GB2 and GB3).**

**
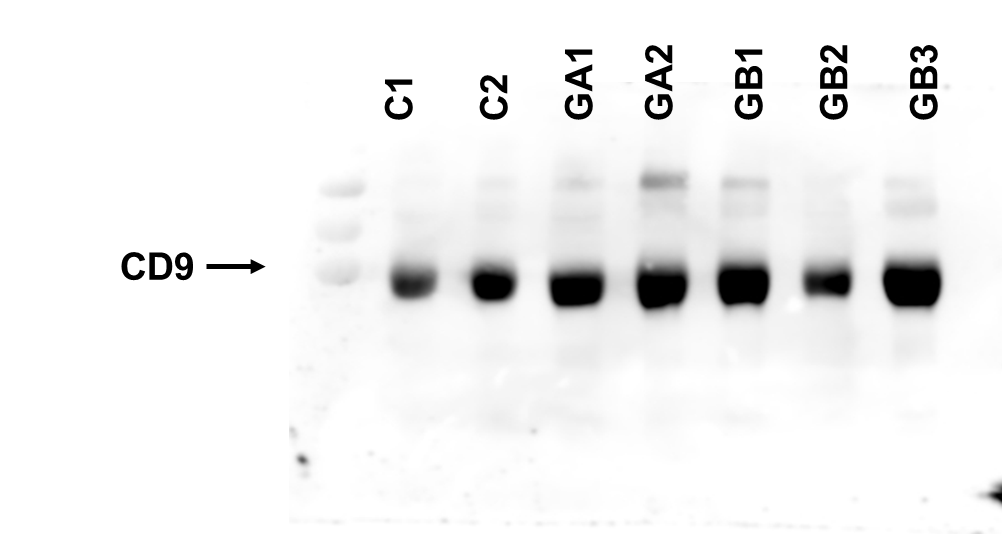
**

**Figure S3: Western Blot of CD9 in Healthy Controls (C1 and C2), Grade I PanNET (GA1 and GA2), Grade II PanNET (GB1, GB2 and GB3).**

**
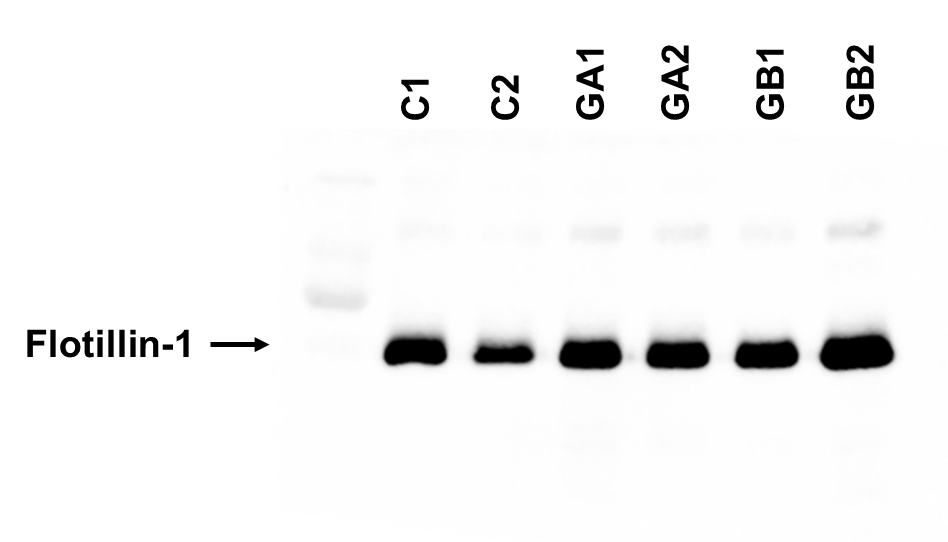
**

**Figure S4: Western blot of anti-Flotillin-1 in Healthy Controls (C1 and C2), Grade I PanNET (GA1 and GA2), Grade II PanNET (GB1, GB2 and GB3).**

**
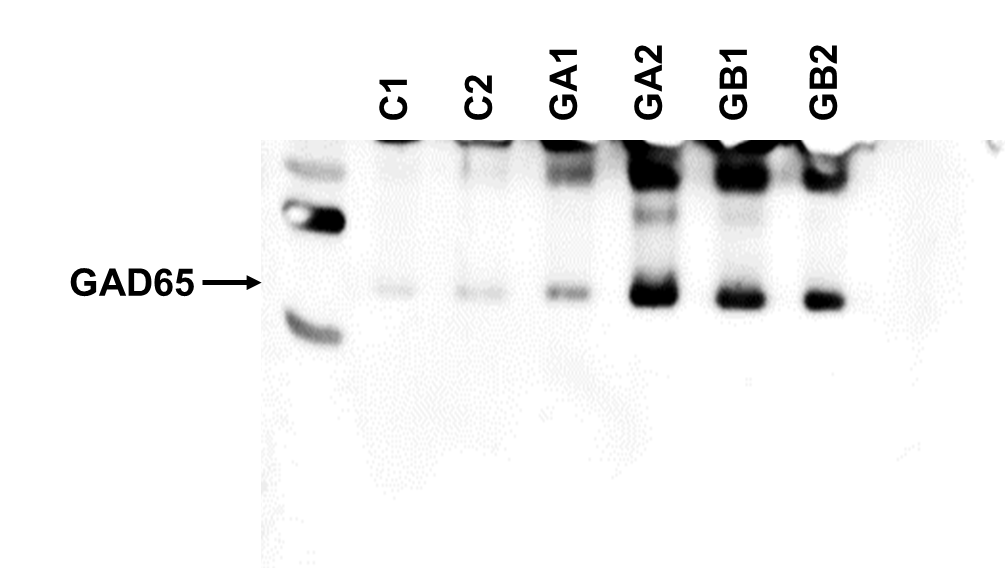
**

**Figure S5: Western blot of anti-GAD65 in Healthy Controls (C1 and C2), Grade I PanNET (GA1 and GA2), Grade II PanNET (GB1, GB2 and GB3).**

**
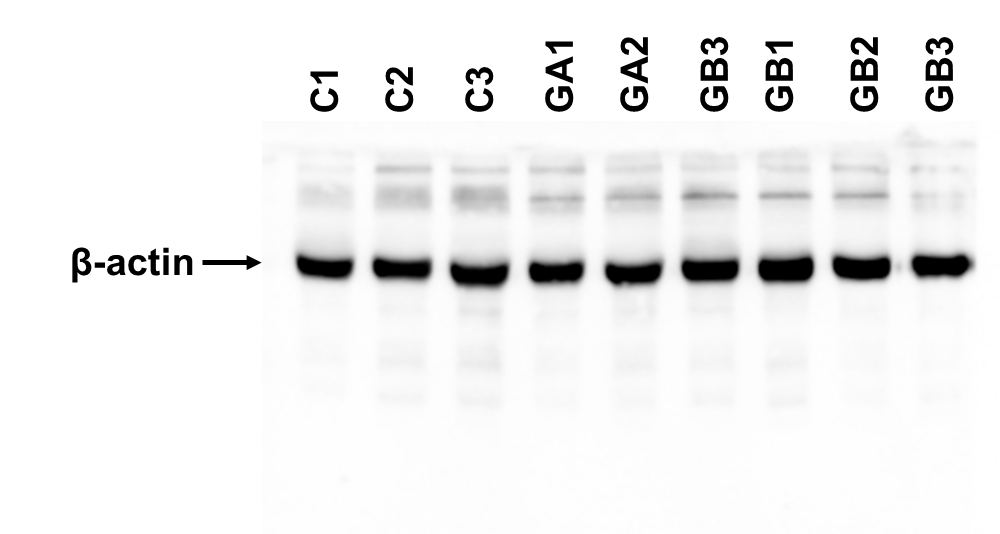
Figure S6: Western blot of anti-β-actin in Healthy Controls (C1, C2 and C3), Grade I PanNET (GA1, GA2 and GA3), Grade II PanNET (GB1, GB2 and GB3).**

**
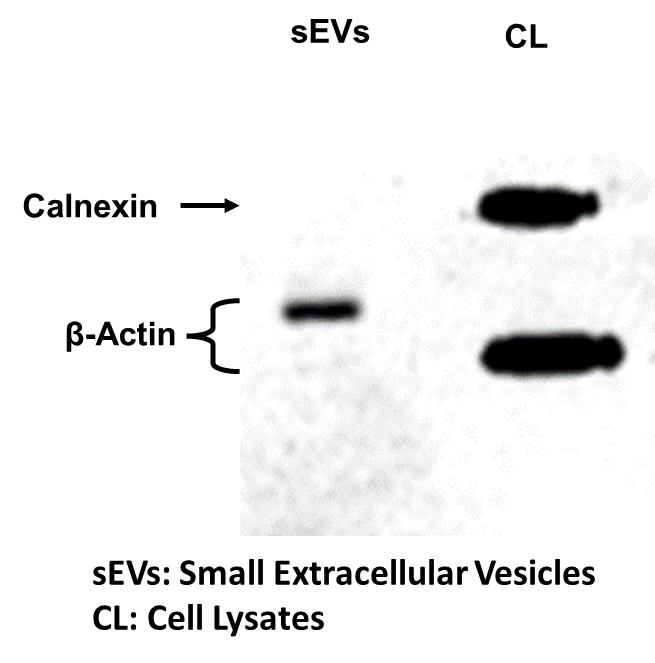
**

**Figure S7: Western blot of anti-Calnexin in sEVs and Cell Lysates.** Calnexin was used to detect non-EV contamination and is a sEV-negative protein marker. CL: cell lysate. Beta-actin was used as a loading control. We observed beta-actin at different molecular weights in sEVs and cell lysates.

**
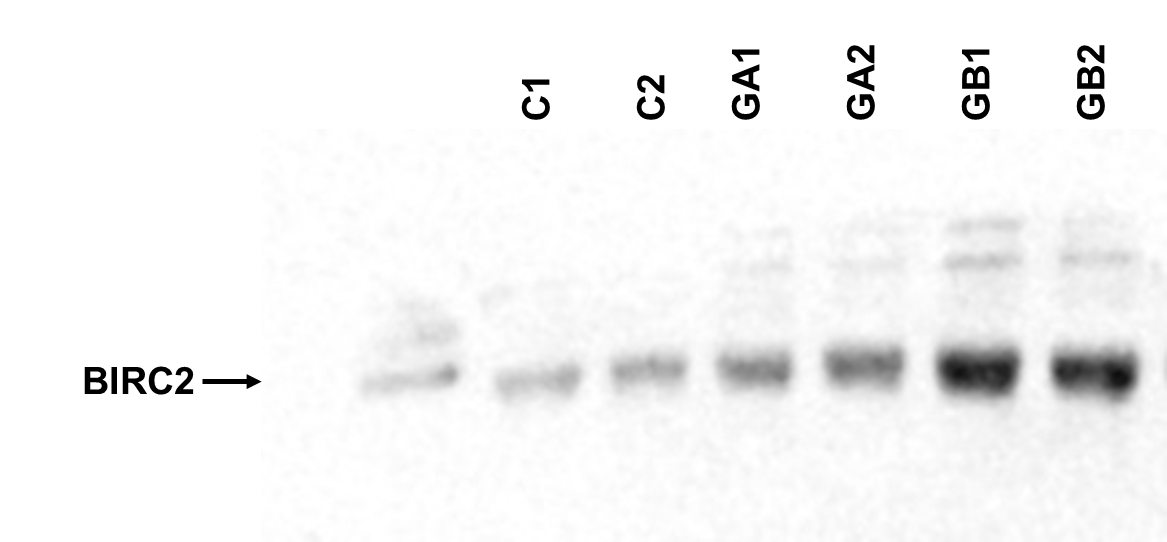
**

**Figure S8: Western blot of anti-BIRC2/cIAP1 in Healthy Controls (C1 and C2), Grade I PanNET (GA1 and GA2), Grade II PanNET (GB1, GB2 and GB3).**


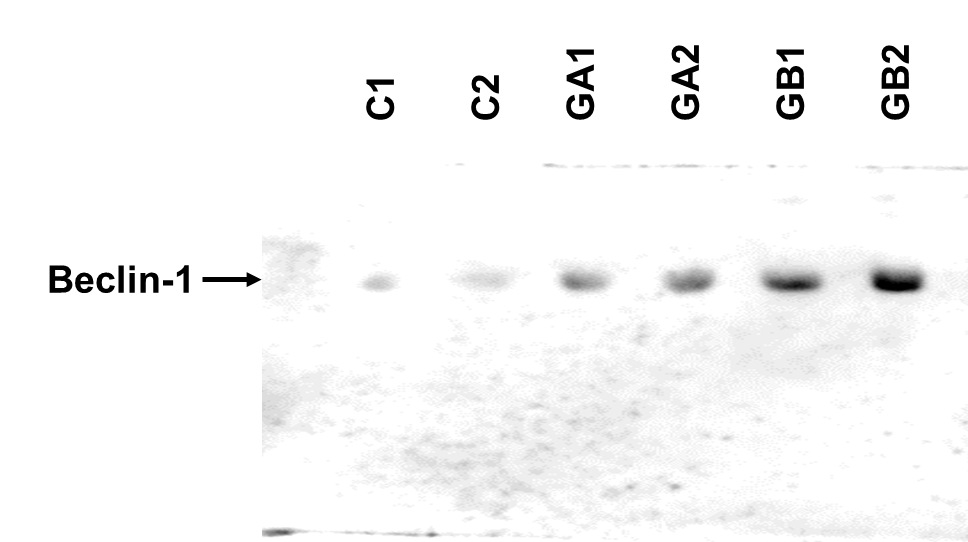


**Figure S9: Western blot of anti-Beclin-1 in Healthy Controls (C1 and C2), Grade I PanNET (GA1 and GA2), Grade II PanNET (GB1, GB2 and GB3).**
